# Cross-Layer-Aided Opportunistic Routing for Sparse Underwater Wireless Sensor Networks

**DOI:** 10.3390/s21093205

**Published:** 2021-05-05

**Authors:** Danfeng Zhao, Guiyang Lun, Rui Xue, Yanbo Sun

**Affiliations:** 1 College of Information and Communication Engineering, Harbin Engineering University, Harbin 150001, China; zhaodanfeng@hrbeu.edu.cn (D.Z.); xuerui@hrbeu.edu.cn (R.X.); 2 Southwest China Institute of Electronic Technology, Chengdu 610036, China; syb12345@hrbeu.edu.cn

**Keywords:** underwater wireless sensor networks, cross-layer routing, opportunistic routing, fuzzy logic, network coding

## Abstract

Underwater wireless sensor networks (UWSNs) have emerged as a promising technology to monitor and explore the oceans instead of traditional undersea wireline instruments. Traditional routing protocols are inefficient for UWSNs due to the specific nature of the underwater environment. In contrast, Opportunistic Routing (OR) protocols establish an online route for each transmission, which can well adapt with time-varying underwater channel. Cross-layer design is an effective approach to combine the metrics from different layers to optimize an OR routing in UWSNs. However, typical cross-layer OR routing protocols that are designed for UWSNs suffer from congestion problem at high traffic loads. In this paper, a Cross-Layer-Aided Opportunistic Routing Protocol (CLOR) is proposed to reduce the congestion in multi-hop sparse UWSNs. The CLOR consists of a negotiation phase and transmission phase. In the negotiation phase, the cross-layer information in fuzzy logic is utilized to attain an optimal forwarder node. In the transmission phase, to improve the transmission performance, a burst transmission strategy with network coding is exploited. Finally, we perform simulations of the proposed CLOR protocol in a specific sea region. Simulation results show that CLOR significantly improves the network performances at various traffic rates compared to existing protocols.

## 1. Introduction

Underwater Wireless Sensor Networks (UWSNs) have been a promising networking technique for marine data collection, pollution monitoring, disaster prevention, assisted navigation, and tactical surveillance applications [1,2,3]. An especially representative application scenario is composed of large amounts of homogeneous nodes with self-organizing, which are deployed in a region of submarine environment [4,5]. In UWSNs, the nodes are sparsely deployed over a large area due to the high manufacturing cost and high design cost [1,6]. Each source node transmits its information to the sink node through multi-hop, and then, the sink node forwards corresponding information to onshore center via RF transmission.

A series of opportunistic routing protocols have been developed for multi-hop sparse UWSNs, since traditional routing protocols cannot work well in underwater scenarios [7,8]. Cross-layer design is an effective approach for optimization of the mechanisms at different layers to accommodate the variability of the characteristics of the underwater channel [1], which were especially required in a UWSN as a stringent constraint network [9,10]. Recently, many cross-layer OR protocols have been designed for UWSNs, where the geographically-based protocol focused beam routing (FBR) and topologically-based protocol channel-aware routing protocol (CARP) are two typical cross-layer OR protocols for sparse UWSNs [5,11]. In UWSNs, the traffic of the sensor nodes varies significantly in a UWSN, since many nodes act as not only source nodes but also relay nodes. The nodes with more neighboring nodes and the nodes in close proximity to the sink nodes may suffer from congestion problem, because more traffic rates occur around these nodes. FBR heavily suffers from the congestion problem when the traffic load increases. Burst transmission is an effective solution to address the congestion problem [5,12]. CARP adopts the burst transmission strategy to resolve the congestion to some extent. These two protocols only perform better in networks with low traffic rates, and the delivery performance of these protocols degrades heavily as the network traffic increases. The schedule-based transmission protocols can effectively handle high traffic rates in UWSNs [13,14]. These protocols are designed merely for the networks with star topology; however, they cannot well adapt to multi-hop UWSNs. Thus, it is necessary to develop a novel cross-layer protocol on the basis of burst transmission for sparse multi-hop UWSNs with various traffic rates.

Considering the long-term channel occupation in the burst transmission strategy. many aspects particularly essential should be taken into consideration, including the selection of the communication pair over a period of time, the setting of time interval of burst transmission, and the introduction of a strategy to avoid the collision problem at high traffic rates. First, negotiation strategy is a valid way to adaptively select a communication pair with the minimum impact on networks. Second, the time interval occupied by burst transmission is related to the average size of packets in the data queue and channel condition. An appropriate burst size should be set according to the congestion state of the network. In addition, random linear packet coding is a valid transmission strategy to achieve link reliability in burst transmission [15]. Lastly, the back-off strategy is an effective method that is on the basis of negotiation strategy to handle the collision problem.

In this paper, a Cross-Layer-Aided Opportunistic Routing Protocol (CLOR) for sparse UWSNs is proposed by using handshake strategy in the negotiation phase and random linear packet coding strategy in the transmission phase. The multifold contributions of this paper are presented as follows.

In the negotiation phase, a hybrid candidate set selection algorithm based on fuzzy logic is presented, which integrates topologically information, link quality, and congestion. In addition, a back-off strategy in CLOR is introduced to alleviate the collision problem.

In the transmission phase, an adaptive transmission strategy based on inter-session network coding is utilized to improve transmission performance. Then, a burst transmission strategy is also adopted to accommodate the networks with various traffic rates. Further, we execute a random linear packet coding by using systematic network coding to improve the transmission reliability.

Lastly, this paper analyzes the effectiveness of negotiation, the effectiveness of burst transmission, and the reliability of adaptive coding strategy. Moreover, the Bellhop ray tracer is used to model the underwater acoustic channel of a specific sea region, and then, the performance of CLOR and typical routing protocols is comprehensively evaluated and compared.

The rest of this paper is organized as follows: In Section 2, the related works with respect to OR protocols are summarized. Section 3 defines network architecture and channel model exploited in this paper. Then, Section 4 introduces the major strategy used in CLOR and describes the process of CLOR. In Section 5, we analyze the major strategies used in CLOR. The performance evaluation and discussion are presented in Section 6. Finally, the paper is concluded in Section 7.

## 2. Relate Works

Traditional routing protocols establish a complete transmission path before forwarding data. However, these protocols are inefficient for UWSNs due to the distinctive nature of the underwater environment. In contrast, OR protocols establish an online route for each transmission, adapting well to time-varying underwater channels. Typical OR protocols are composed of two procedures: Candidate Set Selection (CSS) and Candidate Set Coordination (CSC) [16,17]. The first procedure is responsible for selecting a set of neighbor nodes as potential next-hop forwarders. The second procedure coordinates the transmission between the current sender and next hop forwarder. In this paper, we review recent OR protocols by categorizing the CSS into receiver-side-based, sender-side-based, and hybrid approaches. In addition, we review the cross-layer aided OR protocols in the last categories, since these protocols exploit hybrid approaches to accomplish CSC. 

In receiver-side-based CSS, the receiver decides whether to forward a packet. The node that receives the packet will check whether it belongs to the candidate set by the CSS algorithm. Then, only the nodes in the candidate set perform the candidate set coordination. A typical protocol that has been earlier proposed is vector-based forwarding (VBF) [18]. In VBF, each source node establishes a virtual three-dimensional (3D) pipe toward the sink. The neighbor nodes in the pipe and on the route to the sink will be added to the candidate set. Each candidate node that has received the data packet evaluates the priority according to its location information, and then sends the data packet in the order of priority. A protocol based on VBF, called hop-by-hop vector-based forwarding (HH-VBF), adds more potential forwarders to the candidate set by adjusting the size of pipes [19]. Depth-based routing (DBR) is a typical OR protocol using depth information to divide candidate sets [20]. In DBR, the neighbor nodes with lower depths are added to the candidate set. Energy-efficient cooperative opportunistic routing (EECOR) also determines the candidate set based on the depth information [21]. In addition, it introduces a fuzzy logic-based relay selection scheme to select an optimal next hop. In EECOR, only the energy consumption and packet deliver rate are considered in fuzzy function. To further optimize the candidate algorithm, energy-efficient optimal relay selection (Co-EEORS) selects the next-hop forwarder using physical distance to the sink together with the depth information of each potential forwarder [22]. A coding-aware opportunity routing method for sparse UWSNs (CORS) develops a forwarding with opportunistic coding (FOC) strategy based on network coding [23]; a sliding window-based coding algorithm provides effective coding gains with low coding overhead, and the coding process is triggered by an incoming packet within a valid coding window. For the protocols in this category, the position information is required to be updated periodically, which may be energy inefficient and may degrade the network performance. Due to the fact that the nodes in candidate sets only forward packets rather than being responsible for interacting with the sender, these protocols will suffer from void problems. Moreover, due to the limitation of integrating with contention-based MAC protocol, the hidden terminal problem cannot be effectively resolved. Furthermore, the network performance may degrade heavily in higher traffic rates and in networks with non-uniform topology.

In sender-side-based CSS, only the sender executes the CSS algorithm. The candidate set of each node is established before each transmission by using the history knowledge of the neighboring nodes in the network. In Hydraulic pressure-based anycast routing (HydroCast), the neighbor nodes with lower depths are added into a candidate set according to the candidate algorithm [24]. Then, the candidate set will be divided into many sub-candidates where the node in the same sub-candidate can overhear each other. Lastly, HydroCast calculates the normalized advance of each sub-candidate set to choose an optimal sub-candidate to forward a packet. Void-aware pressure routing (VAPR) adds all the neighboring nodes to the candidate set and uses the depth information of these nodes to define the path direction [25]. Moreover, the routing direction information of each node is updated through periodically broadcasting. Channel-Aware Reinforcement learning-based Multi-path Adaptive routing (CARMA) is a learning-based protocol that uses reinforcement learning framework to select an optimal candidate set [26]. The performance of this protocol is closely correlated to the effectiveness and reliability of the estimation of the network information. For protocols in this category, the void problem can be resolved to some extent. However, the nodes in the candidate set are only responsible for forwarding data rather than interacting with the sender. Thus, the networks suffer from the hidden terminal problem. Furthermore, the network performance is constrained with real-time acquisition of network information due to the requirement of periodically updating information of the neighboring nodes.

In the hybrid approaches of CSS, each node selects the next-hop forwarder by negotiating with the nodes in the candidate set. According to the feedback from these potential forwarders, a sender chooses a valid next hop by using a candidate selection algorithm. Many protocols in this category are developed based on sender-side-based OR protocols, whereas other protocols are developed based on typical receiver-side-based OR protocols. A protocol called GEDAR takes the same strategy to form a candidate set as exploited in HydroCast [16]. In addition, the node that has received the forwarding packet implicitly sets a priority to the current packet as designed in DBR. Moreover, GEDAR resolves the void problem by adjusting the depth of each potential forwarder. Adaptive hop-by-hop vector-based forwarding (AHHVBF) is developed based on typical VBF by adding the neighborhood table constructed in each node [27]. Moreover, a sender dynamically changes the pipe radius according to the feedback of neighbor nodes to bypass void region. Weighting depth and forwarding area division (WDFAD-DBR), based on DBR, uses the depth information of the two-hop node to participate in candidate set selection and to address the void problem [28]. To accomplish hybrid candidate set selection, WDFAD-DBR introduces some mechanisms to divide the forwarding area and to predict neighbor nodes. By introducing negotiation in this approach, these protocols can avoid the void problem as well as resolve the hidden terminal problem. 

The hybrid approach of OR protocols introduced above are designed for the dense underwater environment of a 3D region, whereas most of the recent applications require data collection in sparse UWSNs. Typical cross-layer OR protocols for sparse UWSNs are FBR protocol [11] and CARP Protocol [5,29]. FBR use geographic information to accomplish candidate set selection. FBR divides a candidate set according to the fan section. Furthermore, in case of a sparse network, power control is used to adjust the range of the sector, and then to enlarge the candidate set, enhancing the robustness in harsh underwater environment. FBR can resolve the void problem by ensuring the number of nodes in a candidate set. However, the interference introduced by the power control strategy degrades the transmission performance at high traffic rates. In case of networks with high traffic rates, more packets are buffered at hotspot nodes, resulting in the congestion problem. In contrast, CARP uses hop count information other than position information. In CARP, the neighboring nodes with smaller hop counts are added to a candidate set. Each sender selects an optimal forwarder by considering topology information, link quality, residual energy, and buffer space from the candidate set. Furthermore, CARP supports transmission for a train of data packets whenever possible, significantly improving transmission efficiency. However, CARP still suffers from the congestion problem at high traffic rates for lack of a congestion avoidance mechanism in the negotiation phase. To this end, this paper designs a novel cross-layer OR routing protocol for improving the transmission reliability at high traffic rates in sparse UWSNs. 

This paper is different from our previous paper [23], which is developed for resolving the routing problem in mobile UWSNs scenarios. CORS is an OR protocol-based on receiver-side-based CSS, whereas CLOR uses a hybrid approach of CSS. In addition, a cross-layer design based on the fuzzy logic method is used to select an optimal forwarder. CLOR combines system network coding with a burst transmission strategy to improve the transmission performance. However, the CORS is based on an opportunistic coding strategy, and each time, a forwarder only can send one coded packet. Moreover, link metric, advance to sink, and congestion metric are introduced in CLOR, whereas these metrics are not used in CORS. Thus, this paper is novel protocol compared with CORS. 

## 3. System Model

In this section, we first describe the architecture of an underwater network in this paper. Then, this paper reviews the underwater channel model and derives Packet Error Rate (PER) that is used in the protocol analysis and in the simulation.

### 3.1. Network Architecture

**Figure 1:**
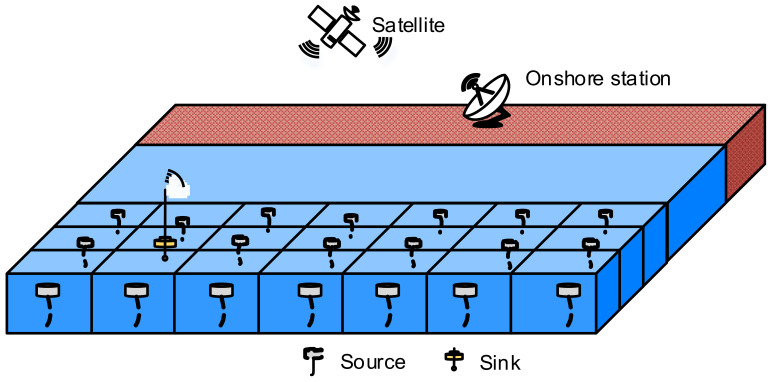
Network architecture.

In this paper, as shown in Figure 1, the network consists of two types of nodes: a sensor node and a sink node. A sensor node is an isomorphic node equipped with a half-duplex omnidirectional acoustic modem and has the capability of collecting, sending, and forwarding information. A sink node is integrated with an acoustic modem receiving information from the sensor nodes and a wireless modem sending information toward a satellite or onshore station. In the networks, each of the nodes are uniformly anchored in different depths of the specific region of the sea. Once sensing information, the source nodes transmit directly or through multi-hop to the sink, which forms many-to-one convergent traffic. Then, the sink will forward the data to the onshore station via the satellite in space [5,30,31].

### 3.2. Channel Model

#### 3.2.1. Energy Propagation Model

In this paper, two propagation models are exploited. First, a theoretical model is used to analyze the effectiveness of control packets. Second, we use the Bellhop model in our simulation, because it has a good approximation of an actual underwater channel environment [32].

Theoretical model: The path loss is related to the distance d and frequency f [33], which can be represented as

(1) $$A(f,d)=A_{p}d^{k}\alpha {\left(f\right)}^{d}$$

where $A_{p}$ is the transmission anomaly that represents the degradation of the acoustic intensity and scattering of sound [34,35,36]. Generally, $A_{p}$ is a log-normally distribution for time-varying shallow water. The parameter k denotes the spreading factor that describes the extended geometric shape (k = 2 for spherical spreading, k = 1 for cylindrical spreading), the absorption coefficient α(f) can be empirically expressed as below [33].

(2) $$10\log_{10}\alpha (f)=0.11\frac{f^{2}}{1+f^{2}}+44\frac{f^{2}}{4100+f}+2.75\cdot 10^{-4}f^{2}+0.003$$

Bellhop model: Bellhop software, based on ray theory, can accurately reflect the attenuation of the underwater channel. We exploit this software to compute the path loss of information and spatially varying interference [32,37]. When using Bellhop, some specific sea information is required, such as SSP, the bathymetric profile, and the type of bottom sediments, which can be obtained from the World Ocean Atlas [38], General Bathymetric Chart of the Oceans [39], and National Geophysical Data Center Deck41 database [40], respectively.

#### 3.2.2. Ambient Noise

The ambient noise of underwater by the empirical formula is described as below [33].

(3) $$N(f)=N_{t}(f)+N_{s}(f)+N_{w}(f)+N_{th}(f)$$

where $N_{t}(f)$, $N_{s}(f)$, $N_{w}(f)$, $N_{th}(f)$ denote the power spectral density (p.s.d) of turbulence, shipping, waves, and thermal noise, respectively. Empirical formulas for these p.s.d in dB re µPa per Hz are given as [33]:

(4) $$\begin{array}{l} 10\mathrm{lg}N_{t}(f)=17-30\mathrm{lg}f\\ 10\mathrm{lg}N_{s}(f)=40+20(s-0.5)+26\mathrm{lg}f-60\mathrm{lg}(f+0.03)\\ 10\mathrm{lg}N_{w}(f)=50+7.5w^{1/2}+20\mathrm{lg}f-40\mathrm{lg}(f+0.4)\\ 10\mathrm{lg}N_{th}(f)=-15+20\mathrm{lg}f \end{array}$$

where the s is the shipping activity factor ranging between 0 and 1; at the same time, the w indicates the wind speed in m/s.

#### 3.2.3. Packet Error Rate

Considering the interference packet, we divide each packet into M parts and then calculate the signal-to-interference plus noise ratio (SINR) of the $m_{th}$ part [23,41,42] as

(5) $$SINR_{m}=\int_{B}\frac{p_{s}(f)/A(f,d)}{N(f)+I_{m}(f)}df,$$

where $p_{s}(f)$ is the power density of the sender. *B* is the total bandwidth in Hz. The $I_{m}(f)$ denotes the superposition of the interference power density in the $m_{th}$ segment, which is evaluated as [23]

(6) $$I_{m}(f)=\sum_{i=1}^{N_{m}}\frac{p_{i}(f)}{A(f,d_{i})}$$

where $N_{m}$ represents the amount of interferences in the $m_{th}$ part of a packet, $d_{i}$ is the distance between a receiver and its $i_{th}$ interference node, and $p_{i}(f)$ is the power density of the $i_{th}$ interference in the $m_{th}$ segment for carrier frequency f. For simplicity, we assume $p_{s}(f)$ as a constant function and $p_{i}(f)=p_{s}(f)$ is the power density of the $i_{th}$ interference within the $m_{th}$ part of a packet. Then, the bit error rate (BER) of the $m_{th}$ segment $p_{b}(m)$ can be calculated by using the BPSK performance function in the AWGN channel of $1/2erfc(\sqrt{SINR_{m}})$ [37,41]. We get the BER of the packet $p_{b}$, which is given by:

(7) $$p_{b}=\frac{\sum_{m=1}^{M}p_{b}(m)l_{m}}{L}$$

where $l_{m}$ represents the length of the $m_{th}$ segment in bytes, and L is the total length of the packet in bytes. 

Finally, we calculate PER of a packet by:

(8) $$PER=1-{\left(1-p_{b}\right)}^{8L}$$

## 4. CLOR Protocol

CLOR is a distributed cross-layer routing protocol, which integrates the candidate set selection algorithm, negotiation, and burst transmission strategy. In this section, we first introduce the main idea in CLOR. Then, the major process of CLOR is exhaustively described.

### 4.1. Main Idea

#### 4.1.1. Forwarder Selection Algorithm

In CLOR, we exploit a hybrid candidate set selection strategy by utilizing metrics such as topological information, link quality, and congestion state.

First, we use hop count information to bypass connectivity holes and shadow zones [5]. The topological information can be obtained through broadcasting of network initialization and be updated in each transmission. Then, we consider the link quality of the forward link to the nodes with low hop count.

Link quality estimation reflects the variation of the time-varying channel. The typical mechanism of a link quality estimator (LQE) can be classified into the hardware-based LQE and software-based LQE [43]. Each node cannot accurately estimate the link quality only by using the Signal-to-Noise Ratio (SNR) due to diverse channel variation. Therefore, we introduce the packet received ratio (PRR) that reflects the link quality at the high layer. SNR can be directly read at the physical layer. PRR can be obtained by using periodic beacons or directly via data traffic rates at the high layer [44].

Second, we exploit the topological information and link quality of multi-hop nodes to avoid routing falls into local optimal [8,45]. On this basis, we calculate the advancement information to the sink.

Third, we introduce the packet received ratio (PRR) that reflects well the link quality at the high layer. Each node measures the local congestion degree (CD) with the arrival rate and the forward rate of the data packets [46]. We consider two types of arrival packets in the network layer; i.e., the data packets from the high layer and the data packets from the other nodes.

These strategies are devoted to resolving different problems that are slightly correlation with each other. Thus, to develop a candidate selection algorithm based on these strategies is a complex problem. Fuzzy logic is a typical method to optimize this problem [21,44]. In this paper, we introduce the fuzzy logic method to functioning these metrics to make forwarding decisions.

#### 4.1.2. Negotiation Strategy

Control-based candidate coordination is an efficient procedure to accomplish hybrid candidate set selection. We employ a handshake before each transmission. However, this strategy suffers from an exposed terminal problem. Carrier sensing is a promising method to resolve this problem [2]. Nevertheless, this problem cannot be well addressed only by using carrier sensing due to the long propagation delay underwater. Since the control packet can be overheard by adjacent nodes, we exploit a novel negotiation mechanism that adopts competition together with carrier sensing in the negotiation phase. In addition, we introduce a back-off strategy in CLOR to alleviate the collision caused by the burst transmission [47].

#### 4.1.3. Coding-Based Burst Transmission Mechanism

As the traffic rate increases, the nodes with more neighboring nodes may buffer more than one packet, increasing the collisions in the network once each node frequently sends a control packet to request to forward a data packet. To resolve this problem, we introduce a burst transmission mechanism. In CLOR, each node can send a train of data packets in the transmission phase. Based on this strategy, the CLOR protocol mitigates the congestion problem and improves the efficiency of negotiation. Considering the varied number of data packets that are buffered in the senders, we adaptively set the burst size in each transmission.

Moreover, the channel of shallow water changes significantly in the networks. Each node should transmit more packets over the burst size to increase the transmission reliability. However, the transmission with a fixed redundancy is inefficient due to the large variations of the channel condition in the network. The sender can encode data without limitation to ensure the reliable recovery of packets at the receiver. However, this type of transmission is costly in terms of energy consumption and limited batteries for a UWSN. Thus, we introduce a packet-level coding strategy based on network coding in the burst transmission process to improve the transmission reliability [15,48,49,50]. The coding redundancy is related to the channel conditions of each forward link. As considered in [43], we estimate the link quality via the feedback of each data transmission. Due to the asymmetry of underwater links, each node is essential to maintain the channel state of bidirectional links.

### 4.2. Process of CLOR

#### 4.2.1. Network Initialization and Maintenance

In the initialization phase, the sink node initializes its hop count information with 0, while the sensor nodes update their hop count information as infinity. Then, the sink establishes and maintains a tree topology by periodically flooding HELLO packets throughout the network [5,22,23]. A HELLO packet contains the unique identifier (ID) of the sink, the hop count (HC) information of the sink, and the stamp time (ST) of this packet. The node that has received a HELLO packet checks whether its own HC is larger than the HC carried by the packet plus 1. If not satisfying the condition, the node discards the packet after updating the neighbor table according to this packet. Otherwise, the node updates its HC by plus 1 to the HC carried in the packet and then rebroadcasts it by inserting its own information into the HELLO packet. This procedure continues until all the remote nodes receives a HELLO packet. After finishing network initialization, every node knows the hop distance from the sink and the topological information of its one-hop neighbors.

The neighbor table in a node includes the ID, the topological information, and the bidirectional link quality of the neighboring nodes. Node degree $D_{x}$ is used to record the number of neighboring nodes of the node x. We add them to both the control packet and data packet to inform other nodes of the variation of topological information and link information. 

#### 4.2.2. Negotiation Process

The negotiation process executes as follows: Before the transmission phase, the sender broadcasts a Request to Forward (RTF) packet to indicate following transmission [3]. Then, each potential forwarder that has received this RTF packet sends back a Clear to Forward (CTF) packet, which contains the progress of the node to act as a next-hop forwarder. Finally, the sender chooses the optimal next-hop forwarder from these nodes. 

We use five distinct states in CLOR: IDLE, WFCTF, WFDATA, WFACK, and BACKOFF. After initialization, each node goes to IDLE state to wait to send data or to receive data. If a node sends an RTF packet, it will go into WFCTF state to wait for the CTF packets for this RTF packet. Similarly, each node goes into WFACK state after sending data packets. When a node has returned back a CTF packet to the sender, it goes into WFDATA state to wait for the following transmission. There is still a situation in which a node does not require any data packets from the sender, since these packets have been received before. For this situation, the node may go into IDLE state after sending a CTF packet. In addition, any node in IDLE state that has received a control packet not for itself may go to BACKOFF state.

In addition to the case that a node receives an invalid RTF packet, there are many cases that a node executes a back-off strategy. For instance, a node that is waiting for a CTF receives a RTF packet whose source has higher competition priority than this node. Any time a node has overheard a CTF for another node, it will execute the back-off strategy. There is an exception in which the node is in the time interval of waiting transmission. The time interval of the back-off strategy is related to the type of the packet and is calculated as below.

(9) $$\tau_{BO}=\left\{\begin{array}{c} 2\cdot \tau_{max\mathrm{Pr}op}+\tau_{PONG},RTF\\ 2\cdot \tau_{max\mathrm{Pr}op}+k\cdot \tau_{D},CTF \end{array}\right.$$

However, there is an exception in which the CTF indicates that none of the data packets is required for transmission. Then, the node should merely ignore the CTF packet.

To alleviate the exposed problem, the carrier sensing is applied before each handshake. If the received power is higher than the preset threshold, the node will cancel the current negotiation. Otherwise, it will execute the handshake immediately. If a node receives an RTF packet from another node while waiting for the CTF packet, it will compete transmission by comparing with the competition information in the RTF packet. If the node fails in the competition, it will back off according to the information in the RTF packet. Otherwise, it will continually execute the negotiation.

The detail process of negotiation is as follows. 

Upon receiving a service request, the node senses the channel. If the channel is not busy, the node acts as a sender and broadcasts an RTF. This packet contains the topological information of the current sender and the information of data packets in burst transmission. Then, the sender sets a timer to wait for the response of potential next-hop nodes.

Once it has received an RTF packet, the node executes Algorithm 1: First, the node updates its neighbor table and its own topological information according to the RTF packet (line 1). Then, it executes the corresponding process according to the state.

If the node is in WFCTF state, it will execute the competition by using the degree information and queue size in the RTF. The node with a lower node degree and small queue size cancels the negotiation and executes the back-off strategy (line 4). Otherwise, if the node is in another state rather than in IDLE state, the node will ignore this negotiation (line 8). 

When the node is in IDLE state, it participates in negotiation. If the RTF comes from a node with smaller hop count than that of itself, the node discards the RTF packet and executes the back-off strategy (lines 10). Otherwise, the node checks the receiving status of the pending data packets in the sender side. The node uses a request mask $M_{rq}$ to indicate the data packets that are requested to be sent. Since the RTF conveys a series of information for the data packets of burst transmission, the node sets the bit field value of the position in $M_{rq}$ to 1 if it requires the data packet (line 13). If at least one packet is requested to be sent, the node executes the candidate set selection algorithm by using the fuzzy logic method (line 15). If all the data packets in the burst transmission have been received, the node will skip this algorithm and set the output value of fuzzy logic $F_{prg}$ to zero. Then, it returns back a CTF packet that contains $M_{rq}$, $F_{prg}$, and the topological information of itself (line 17). Meanwhile, the node sets a timer to wait for the data packets according to the bit field value of $M_{rq}$. If all the bit field values of $M_{rq}$ are zeros, the node ignores the following transmission and goes back to IDLE state.
**Algorithm 1** Upon receiving an RTF packet $P_{RTF}$1: updates neighbor table and topological information. 2: **if** (State=WFCTF) **then** 3:  **if** ($N_{Q}(x)\leq N_{Q}(RTF)\&\&D_{x}<D_{RTF}$) **then** 4:   back off according to (9) 5:  **end if** 6:  discard $P_{RTF}$ 7: **else if** (State≠IDLE) **then** 8:  discard $P_{RTF}$ 9: **else if** ($y\notin C_{x}$) **then** 10:   back off according to (9) 11:   discard $P_{RTF}$ 12:  **else** 13:   set $M_{rq}$ according to the burst information in $P_{RTF}$; 14:   **if** ($bitsum(M_{rq})\neq 0$) **then** 15:   execute fuzzy logic 16:   **end if** 17: create a CTF packet and send it to transmit queue. 18: **end if**

If it has not received any reply when the timer expires, the node restarts a new negotiation until the retry times reach the maximum value. Once receiving a CTF, the sender updates topological information, link quality, and requesting mask. We define the mask from the $i_{th}$ CTF packet $M_{rq}(i)$ as below.

(10) $$M_{rq}(i)=\sum_{j=1}^{N_{BT}}b_{rq}(i,j)x^{j}$$

where $b_{rq}(i,j)$ is the $j_{th}$ bit field value of the require mask in the $i_{th}$ CTF packet, and $N_{BT}$ is the number of original data packets in the burst transmission.

In addition, to improve the transmission effectiveness at high traffic rates, this paper introduces a minimum value of burst size $N_{mBT}$. A negotiation can be triggered only in case that the number of packets in the data queue is higher than or equal with the threshold $N_{mBT}$. In addition, to avoid that a transmission occupies a channel for a long time, a maximum value $N_{MBT}$ needs to be set. The number of the outgoing original packets should not be larger than $N_{MBT}$.

As the waiting timer expires, the node calculates a transmission mask $M_{tx}$ as below.

(11) $$M_{tx}=\sum_{j=1}^{N_{BT}}\left(\prod_{i=1}^{N_{ctf}}b_{rq}(i,j)\right)x^{j}$$

where $N_{ctf}$ is the number of CTF packets that has been received by the sender. Then, the sender checks the bit field in $M_{tx}$. If all the data packets in transmission queue have been received by these nodes in the candidate set, the sender will remove these data packets in the queue and cancel the following transmission. Otherwise, the sender executes the candidate set selection algorithm to calculate the priority of forwarding as below.

(12) $$F_{c}(i)=\omega_{c}\cdot F_{prg}(i)+(1-\omega_{c})\frac{1}{hc_{i}}$$

where $hc_{i}$ is the hop count of the $i_{th}$ node in the candidate set, $F_{prg}(i)$ is the output value of the fuzzy logic that represents the progress of the $i_{th}$ node, and $w_{c}\in [0,1]$ is a weight factor. 

Finally, the sender selects the node with the highest value of $F_{c}(i)$ as an optimal forwarder.

#### 4.2.3. Forwarder Selection Algorithm

##### Fuzzy Input

Generally, *SNR* is estimated based on hardware that can be obtained from the physical layer. Due to the channel variation, an average *SNR* (*ASNR*) as a valid input of fuzzy logic is considered. We calculate *ASNR* by computing the average value of the latest M packets in the link as below.

(13) $$ASNR=\frac{1}{M}\sum_{i=N-M+1}^{N}SNR(i)$$

where N is the total number of packets that are received in the link. M is related to the channel status.

*PRR* is defined by the number of successfully received packets at the receiver divided by the total number of packets sent by the sender. We calculate it at the network layer and use exponential window moving average (EWMA) to estimate Smooth *PRR* (*SPRR*) as below.

(14) $$SPRR(\alpha ,w)=\alpha \cdot PRR_{c}+(1-\alpha )\cdot SPRR$$

where $PRR_{c}$ is the *PRR* of current transmission, α∈[0,1] is the moving factor, and w is the length of the window that is used for link estimation. Here, we take α=0.1 and w=2 in the simulation.

In addition, we introduce the advance to sink (*ATS*) that reflects the delivery ratio of next hop to the sink, which is defined as below.

(15) $$ATS=1-\prod_{i=1}^{N_{LHC}}\left(1-PRR_{i->j}\right)$$

where $N_{LHC}$ is the number of neighboring nodes with values of *HC* smaller than that of the sender, and $PRR_{i->j}$ is the packet received ratio of the $i_{th}$ node in the candidate set. This metric is different from [51], where each time, only one forward link is considered in forwarding in an opportunistic network. However, all the next forward links of the candidate node are considered in this paper.

In addition, to measure the local congestion state, we introduce the congestion degree (*CD*), which is defined as below [46].

(16) $$CD=\frac{\tau_{fw}}{\tau_{arr}}$$

where $\tau_{fw}$ is the average value of the time interval for forwarding a packet, and $\tau_{arr}$ is the average value of the time interval for arriving a packet. When *CD* is higher than 1, the arrival rate is higher than the forwarding rate, and the network suffers from congestion.

##### Fuzzy Rules

We calculate the member functions of $\mu_{ASNR}$, $\mu_{PRR}$ and $\mu_{ATS}$ by using a piecewise linear form function of $\mu_{1}(x)$ and calculate the member function of $\mu_{CD}$ by using function with a form of $\mu_{2}(x)$. The parameters [*a*, *b*] of the member functions in our simulation are set as [1.0 dB, 10 dB], [0.15, 0.95], [0.0, 1.0], and [0.1, 1.0] for the *ASNR*, *PRR*, *ATS*, and *CD,* respectively.

(17) $$\mu_{1}(x)=\left\{\begin{array}{c} 0,x<a\\ \frac{x-a}{b-a}\\ 1,x\geq b \end{array}\right.,a\leq x<b$$

(18) $$\mu_{2}(x)=\left\{\begin{array}{c} 1,x<a\\ \frac{x-a}{b-a}\\ 0,x\geq b \end{array}\right.,a\leq x<b$$

Then, the output value of fuzzy logic is mapped according to the fuzzy rule as follows:

**IF** the forward link has high channel quality **AND** the forward link has high packet delivery ratio **AND** the node has high advance to the sink **AND** the node has low congestion degree, **THEN** it has high priority.

We use Yager AND-like and OR-like operators to produce the value of progress for its compensatory [44,52].

(19) $$\begin{array}{l} F_{prg}=\beta \cdot \min(\mu_{1}(ASNR),\mu_{1}(SPRR),\mu_{1}(ATS),\mu_{2}(CD))\\ \;\;+(1-\beta )\cdot \mathrm{mean}(\mu_{1}(ASNR),\mu_{1}(SPRR),\mu_{1}(ATS),\mu_{2}(CD)) \end{array}$$

where $\beta \in \left[0,1\right]$ is the weight factor, which is related to the network model and the deployment environment. Here, we set β as 0.9 in this simulation.

#### 4.2.4. Adaptive Transmission Process

**Figure 2:**
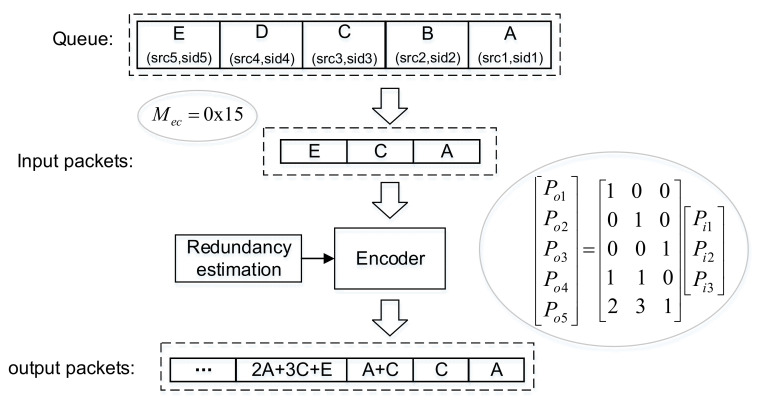
An example of burst transmission based on network coding.

After choosing an optimal forwarder from the candidate set, the sender executes burst transmission, as explained in Figure 2. First, the sender uses encode mask $M_{ec}$ to filter out the invalid data packets in the data queue. Second, the sender estimates transmission redundancy by using forward link quality, and then, it calculates the number of coded packets. Third, the sender generates a random coding matrix and then executes random linear packet coding for these input packets. Lastly, the sender puts the output packets to the transmit queue.

Generally, encode mask $M_{ec}$ is the same with the data transmission mask $M_{tx}$ in the negotiation process and can be represented as below.

(20) $$M_{ec}=\sum_{i=1}^{N_{BT}}b_{ec}(i)x^{i}$$

where $b_{ec}(i)$ = 1 represents the $i_{th}$ packet in the queue that is valid to participate in coding. Then, the number of input packets $P_{I}$ for coding $N_{in}$ can be calculated by

(21) $$N_{in}=\sum_{i=1}^{N_{BT}}b_{ec}(i)$$

Then, we calculate the number of coded packets $N_{out}$ as below.

(22) $$N_{out}=\left\lceil \lambda_{ec}\cdot N_{in}/PRR_{i\to j}\right\rceil$$

where $\lambda_{ec}$ is the redundancy factor for coding, and $PRR_{i\to j}$ is the packet reception ratio of the forward link coming from the sender i to the receiver j.

The sender executes systematic network coding by using coding matrix $G_{s}={\left[I|G_{0}\right]}^{T}$ [53] where I is an identity matrix with a size of $N_{in}\times N_{in}$. matrix $G_{0}$ is generated randomly in Galois field, the size of which is $(N_{out}-N_{in})\times N_{in}$. Then, we can obtain the output of coding $P_{O}$ as below.

(23) $$P_{O}=G_{s}\cdot P_{I}$$

The receiver only knows the original information of these data packets; however, we have no knowledge about the input information and output information of this coding. There are two ways to transmit the coding information: one way is to add the coding vector in the output packet and the other way is to store the coding matrix locally. In CLOR, we add all the coding information in the coding vector $V_{ec}$. Then, other nodes in the candidate set that overhear sufficient coded packets can attempt to recover the original packets. $V_{ec}$ includes the index and the coefficients of the input packets. In addition, the information of burst transmission $M_{ec}$, $N_{out}$, and the index of the output packet EoIdx are introduced to each output packet. Thus, the receiver sets a valid time interval according to this information for waiting for the rest of the data packets in this transmission.

As shown in Figure 2, we use an example to illustrate the coding process: after filtering the invalid data packets in the queue, the node obtains three input packets $P_{I}={\left[P_{i1},P_{i2},P_{i3}\right]}^{T}$, and then, the node can calculate $N_{out}$= 5 by Formula (22). On this basis, the coding matrix is generated to encode the input packets $P_{I}$. Finally, the coded packets $P_{O}={\left[P_{i1},P_{i2},P_{i3},P_{i1}+P_{i2},2P_{i1}+3P_{i2}+P_{i3}\right]}^{T}$ are obtained after executing the coding algorithm according to Formula (23). 

After finishing coding, the node adds these output packets into the transmit queue. Meanwhile, the sender sets a timer to wait for the acknowledgement (*ACK*) packet from the receiver. The time interval of the timer $\tau_{wACK}$ is calculated as:

(24) $$\tau_{wACK}=2\cdot \tau_{max\mathrm{Pr}op}+N_{out}\cdot \tau_{ED}$$

where $\tau_{max\mathrm{Pr}op}$ is the maximum propagation delay of one hop and $\tau_{ED}$ is the transmission delay of a coded data packet.

Upon receiving a new coded packet, the receiver executes On the Fly Gaussian Elimination (OFG) to decode the information [54,55]. The decoding process goes on until the timer for decoding is expired. The receiver sets an initial timer and sends an *ACK* packet back to the sender. The sender may send the packets, and the number of the packets can be smaller than the number required by the receiver, since some packets have been received by other nodes before current transmission. In addition, the receiver cannot accurately estimate the transmission redundancy of the sender. Thus, once a new packet is received, the receiver refreshes the timer according to the coding information in this packet. The time interval of this timer is calculated as follows.

(25) $$\tau_{sACK}=\left\{\begin{array}{c} R_{M}\cdot N_{rq}\cdot \tau_{ED}+\tau_{est},n_{r}=0\\ ((N_{out}-1)-k_{r})\cdot \tau_{ED},n_{r}>0 \end{array}\right.$$

where $n_{r}$ represents the number of the packets that have been received from the sender; and $R_{M}$ is the maximum transmission redundancy initialized by the receiver. $N_{rq}$ is the number of the data packets requested by the receiver; $\tau_{est}$ is the estimated time for the receiver receiving a data packet, which is related to the time interval of current negotiation; $k_{r}$ is the index of the latest coded packet that has been received. On this basis, the time interval $\tau_{sACK}$ is estimated by the number of residual packets.

When the timer is expired, the receiver sends an *ACK* packet that carries the link quality and the decoding results. Meanwhile, the receiver updates the link quality by current transmission and refreshes the neighbor table. When the sender has received an *ACK* packet, it removes the packets according to the decoding mask in the *ACK* packet and then updates the forward link quality.

Any node in the candidate set that has overheard the coded packet attempts to recover the original data packets by the currently accumulated information. The recovered packets are stored in the data queue for future transmission or for protection, since more than one node stores the same data packets. 

## 5. Protocol Analysis

In this section, we analyze the main mechanism exploited in our design. First, the effectiveness of negotiation is derived; then, the reliability of the coding strategy is analyzed.

### 5.1. Effectiveness of Negotiation

The effectiveness of negotiation is related to multi-hop interferences. However, the interference range $R_{i}$ is large underwater. Additionally, the noticeable ambient noise increases the demand of high transmission power. Considering these factors, we derive the relational expression of effectiveness of negotiation $E_{ngt}$ as below.

Considering the network scenario where the nodes are sparsely deployed in shallow underwater and the transmission range $R_{tx}$ is much larger than the depth of the sea region, we use the two-dimension network model to analyze $E_{ngt}$. To facilitate analysis, we use d to represent the distance between sender *S* and forwarder *R*.

**Figure 3:**
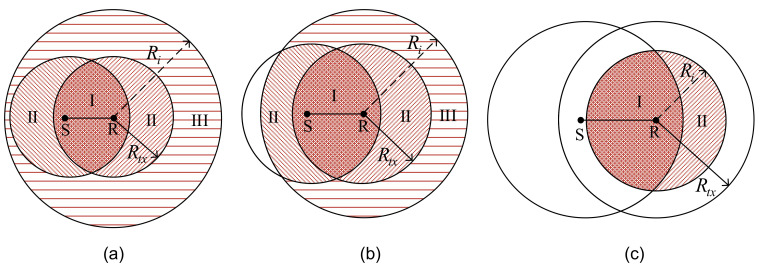
The relationship between the negotiation region and interference region: (**a**) Scenario 1: $0<R_{tx}\leq (R_{i}-d)$; (**b**) Scenario 2: $(R_{i}-d)<R_{tx}<R_{i}$; (**c**) Scenario 3: $R_{tx}\geq R_{i}$.

As shown in Figure 3, we classify all the situations into three categories with three relationship between $R_{tx}$ and $R_{i}$ [56,57,58,59].

As shown in Figure 3, interference region in Scenario 1 contains three zones. In zone I, the interference nodes can overhear both RTF and CTF packets. In zone II, the interference nodes only can overhear RTF or CTF. In zone III, any control packets cannot be overheard correctively. We divide the interference region of Scenario 2 into three zones as well. In Scenario 3, the interference region contains two zones. Then, $E_{ngt}$ can be expressed as:

(26) $$E_{ngt}=\frac{S_{I\cap (ngt)}}{S_{I}}$$

where $S_{I}$ represents the area of the region with interferers, $S_{ngt}$ denotes the area of the region controlled by negotiation control packets, and $S_{I\cap (ngt)}$ is the intersection of the area of these two regions.

**Figure 4:**
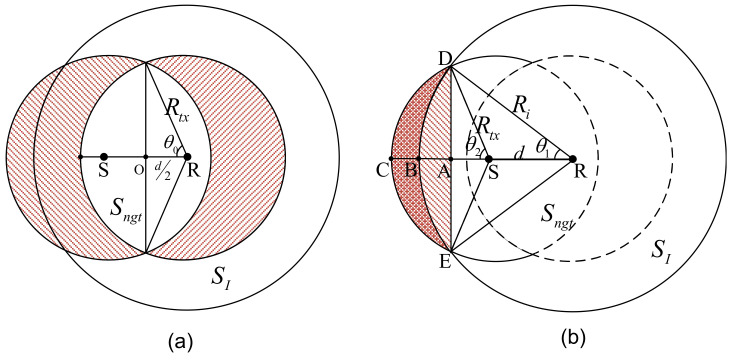
The area of region $S_{I\cap (ngt)}$ in different scenarios: (**a**) Scenario II; (**b**) Scenario III.

We introduce $R_{i}=\gamma d$, where *γ* is spatial reuse factor. Moreover, we use $R_{tx}=d/\lambda$ to represent $R_{tx}$. Referring to the process of [59], we derive the expression of $E_{ngt}$ as follows:

(27) $$E_{ngt}=\left\{\begin{array}{l} \frac{2\pi -2\theta_{0}+\lambda \sin(\theta_{0})}{\pi \lambda^{2}\gamma^{2}},\frac{1}{\left(\gamma -1\right)}\leq \lambda \leq 1\\ \frac{4\pi -4\theta_{0}+2\lambda \sin(\theta_{0})+2\theta_{1}\lambda^{2}\gamma^{2}-\sin(2\theta_{1})\lambda^{2}\gamma^{2}-2\theta_{2}+\sin(2\theta_{2})}{2\pi \lambda^{2}\gamma^{2}},\frac{1}{\gamma }<\lambda <\frac{1}{\left(\gamma -1\right)}\\ 1,\;0<\lambda \leq \frac{1}{\gamma } \end{array}\right.$$

where angles $\theta_{0}$, $\theta_{1}$, and $\theta_{2}$ are shown in Figure 4 and are represented as follows.

(28) $$\left\{\begin{array}{l} \theta_{0}=\arccos(\frac{d}{2R_{tx}})\\ \theta_{1}=\arccos(\frac{d^{2}+R_{i}^{2}-R_{tx}^{2}}{2dR_{i}})\\ \theta_{2}=\arccos(\frac{R_{i}^{2}-d^{2}-R_{tx}^{2}}{2dR_{tx}}) \end{array}\right.$$

The spatial reuse factor γ is related to the frequency and distance. We firstly use the formula of receiver detection threshold T given in [56] to derive the spatial reuse factor γ(f,d).

(29) $$T=\frac{P_{s}/A(f,d)}{P_{i}/A(f,d_{i})+\sigma_{N}(f)}$$

The γ(f,d) can be calculated as below.

(30) $$\gamma^{-k}{\left(\alpha {\left(f\right)}^{d}\right)}^{1-\gamma }=\frac{P_{s}}{P_{i}T}-\frac{A_{p}\sigma_{N}(f)}{P_{i}d^{-k}\alpha {\left(f\right)}^{-d}}$$

where $P_{s}$ is the transmit power of the node *S*, and $P_{i}=P_{s}$ is the transmit power of node *R*, since we assume that each node has the same transmit power.

As a result of no closed solution for γ(f,d), we solve this equation numerically. On the basis of BER = $10^{-4}$, the detection threshold with modulation of BPSK in the Gaussian channel is corresponding to 8.4 dB. Considering the scale factor σ = 4 dB that represents the receiver inefficiencies [37], thus, we set T = 12.4 dB. The central frequency is set to 25.6 kHz. We set *k* =1.75 to give a better asymptotic of the Bellhop model used in the simulation. In addition, we assume that the distribution of $A_{p}$ is $10\times \mathrm{lg}(A_{p})\sim N(10,\delta_{p}{}^{2})$.

**Figure 5:**
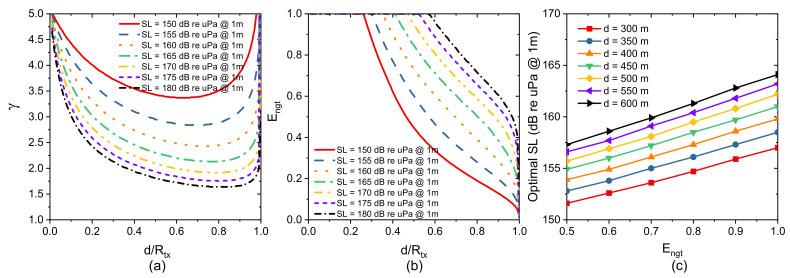
(**a**) Reuse factor γ(f,d) varies with normalized node separation $d/R_{tx}$; (**b**) Effectiveness of negotiation $E_{ngt}$ varies with normalized node separation $d/R_{tx}$; (**c**) Optimal SL for different effectiveness of negotiation $E_{ngt}$.

Figure 5a shows the relationship between the spatial reuse factor γ and normalized node separation $d/R_{tx}$ for several SL. γ decreases as SL increases and reaches a certain value with high values of SL. In addition, spatial reuse decreases as $d/R_{tx}$ increases until that $d/R_{tx}$ reaches 0.7, especially with high SL. In [59], we can obtain γ with a value smaller than 2 when we set SL = 155 dB; however, we require a high value of SL in this paper, since we consider σ and $A_{p}$ in a network scenario. In a real ocean scenario, more transmit power is required to obtain a small value of γ. To improve the spatial reuse of a network, we should find an appropriate SL that is large enough to ensure the connectivity of network topology and that is small enough for approaching a high value of $d/R_{tx}$.

We plot $E_{ngt}$, which varies with $d/R_{tx}$ for different values of SL, in Figure 5b. $E_{ngt}$ decreases as distance d increases with a fixed $R_{tx}$. Moreover, $E_{ngt}$ can be improved by increasing the transmit power. When SL is set to 180 dB uPa @1m source, $E_{ngt}$ can reach 100% when $d/R_{tx}$ is higher than 0.57, and this value is smaller than that in [59], where the threshold value is 0.74. This implies that scale factor σ and transmission anomaly $A_{p}$ heavily influence the effectiveness of negotiation.

To give a comprehensive analysis, we plot the optimal SL for different negotiation efficiency at a fixed distance d in Figure 5c. To ensure the 100% effectiveness of negotiation strategy for d = 300/600 m, SL should be larger than 157/164 dB in relation to uPa @1m source. Similarly, to obtain 80% effectiveness of the negotiation strategy, we have to set SL to a value higher than 154.7/161.3 dB re uPa @1m source for d = 300/600 m. Considering the effectiveness of the negotiation strategy, we should set an appropriate source level for a specific network scenario to improve the network performance.

### 5.2. Effectiveness of Burst Transmission

The burst transmission strategy can be effective at improving the transmission at the hot terminal that buffers more packets or in networks with higher traffic rates. We compare the normal transmission and the burst transmission strategy as below.

We calculate the average waiting time for transmission of the data packets in the queue of a node as below.

(31) $$\tau_{wNT}=\frac{1}{N_{BT}}\sum_{i=1}^{N_{BT}}\left(\tau_{buf}(i\right)+i\cdot \tau_{ngt}+(i-1)\tau_{trans}+\tau_{cmpt}(i-1)))$$

where $\tau_{buf}(i)$ is the buffering time of the $i_{th}$ data packet in the queue and $\tau_{cmpt}(i)$ is the time interval of the $i_{th}$ transmission competition; $\tau_{D}$ is the transmission delay of a data packet; $\tau_{ngt}$ and $\tau_{trans}$ are the time intervals of the negotiation phase and the transmission phase, which are calculated as:

(32) $$\left\{\begin{array}{l} \tau_{ngt}=\tau_{R}+\tau_{C}+2\tau_{p}\\ \tau_{trans}=\tau_{D}+\tau_{A}+2\tau_{p} \end{array}\right.$$

where $\tau_{R}$ and $\tau_{C}$ are the transmission delays of an RTF packet and a CTF packet, respectively; $\tau_{p}$ is the propagation delay between the sender and the receiver; and $\tau_{A}$ is the transmission delay of an *ACK* packet.

When using the burst transmission strategy, it can have a shorter average waiting time as below.

(33) $$\tau_{wBT}=\frac{1}{N_{BT}}(\sum_{i=1}^{N_{BT}}(\tau_{buf}(i)+(i-1)\tau_{D})+\tau_{ngt})$$

We use ${\bar{\tau }}_{cmpt}$ to represent the average time interval of competition for the transmission. Then, the difference between the average transmission times of these two strategies can be represented as:

(34) $$\Delta \tau_{wTX}=\tau_{wNT}-\tau_{wBT}=\frac{N_{BT}-1}{2}(\tau_{ngt}+\tau_{trans}+\tau_{cmpt}-\tau_{D})$$

The burst transmission strategy improves the effectiveness of transmission but introduces network overhead. The selection of burst size $N_{BT}$ influences the transmission performance. If $N_{BT}$ is set to a small value, many packets queued in a node cannot be sent in time. In contrary, if a large value of $N_{BT}$ is set, the channel is occupied by a transmission pair over a long time, and the unequal distribution of transmission in the network may degrade the network performance. So, $N_{BT}$ should be adaptively set for each transmission. 

### 5.3. Reliability of Network Coding

Network coding introduces coding overhead due to the coding matrix and the control information for coding. In systematic network coding, the overhead of coefficient matrix $G_{s}$ can be calculated as below [60].

(35) $$S_{coeffMatrix}=\sum_{n=1}^{N_{MBT}}n^{2}\cdot q$$

The coding overhead $R_{ec}$ has two parts: the first part is coding parameters $N_{out}$, EoIdx, and mask $M_{ec}$; the second part is coding coefficients. We calculate $R_{ec}$ as:

(36) $$R_{ec}=8\cdot \left\lceil (N_{MBT}+2\log_{2}((\lambda_{M}+1)N_{MBT})+N_{MBT}\cdot q)/8\right\rceil$$

where $\lambda_{M}$ represents the maximum transmission redundancy. 

**Figure 6:**
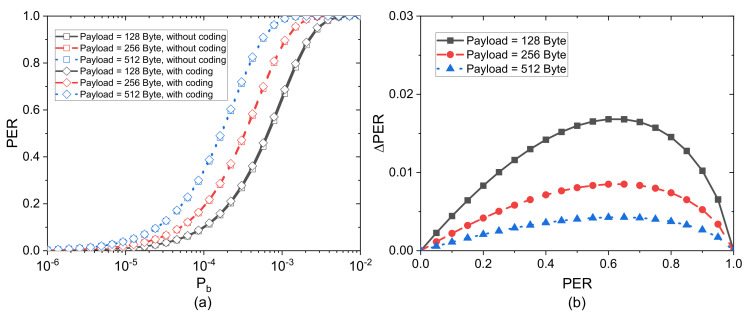
(**a**) *PER* of transmission with and without coding versus $P_{b}$ for different payload sizes; (**b**) the difference of *PER* (Δ*PER*) varies with *PER* for different payload sizes.

Using Formula (8), we calculated the *PER* of different payload sizes with and without coding. Figure 6a shows the *PER* as a function of $P_{b}$ for different packet sizes in the case that $N_{MBT}$ is 16, $\lambda_{M}$ = 2, and GF(8). When the packet size is large, the *PER* varies slightly for different $P_{b}$. We can find that the *PER* only varies slightly for different $P_{b}$ in case of typical packet size. Then, we use $\Delta PER=PER_{ec}-PER_{normal}$ to represent the difference of *PER* after adding coding redundancy to a coded packet. Figure 6b shows that the ΔPER varies for different *PER*. As the channel lightly worsens, ΔPER increases when *PER* is smaller than 0.6. As *PER* increases continuously, ΔPER decreases with the increase of *PER*. Thus, the coding redundancy lightly affects the link quality.

To analyze the decoding performance of the Systematic Network Coding (SNC), we define the Packet Loss Rate (PLR) as the ratio of the number of packets lost at next-hop forwarders to the number of packets from the sender. We compare its PLR performance with that of Opportunistic Routing (OR) and Network Coding (NC) in two typical scenarios: (a) one forward link and (b) two forward links. We set the transmission redundancy $\lambda_{M}$ to 1.0, the burst size $N_{BT}$ with 16, and over Galois fields GF(2), GF(8), and GF(16).

**Figure 7:**
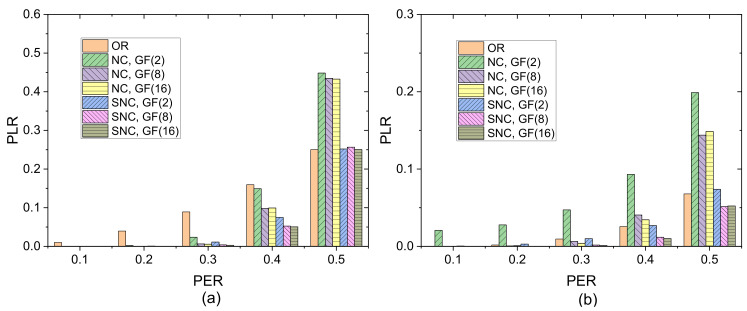
PLR of three strategies versus PER in different Galois fields when $\lambda_{M}$ = 1.0: (**a**) one forward link; (**b**) two forward links.

Figure 7 shows PLR versus PER for three strategies in different Galois fields when transmission redundancy $\lambda_{M}$ is set to 1.0. When PER is small, all three mechanisms can nearly recover all the packets with high probability. As PER increases, the PLR performance of OR degrades heavily, while NC and SNC outperform NR on one forward link. In contrast, in the case of two forward links, OR shows better performance than NC while SNC remain maintains better performance than the other two transmission strategies. In addition, the performance of SNC degrades slightly over the other two strategies as PER increases.

**Figure 8:**
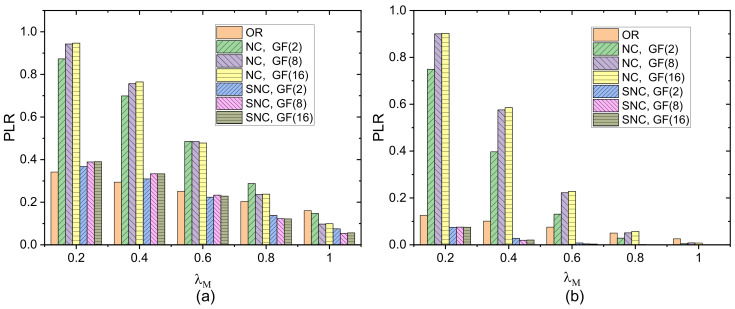
PLR of three strategies versus transmission redundancy in different Galois fields when *PER* = 0.4: (**a**) one forward link; (**b**) two forward links.

Figure 8 shows the PLR of the three strategies versus transmission redundancy for different Galois fields when *PER* = 0.4. NC shows the worst performance compared with the other two strategies when the redundancy is small. The lack of coded packets with a small degree results in low decoding probability, since sufficient coded packets with NC to recover original data packets are required. In contrast, the more coded packets with degree 1 in SNC, the higher the decoding probability, and the performance of SNC is comparable to that of OR. As the redundancy increases, SNC outperforms the other two strategies. As can be seen from Figure 7 and Figure 8, the performance of SNC with GF(8) is close to that of GF(16), and it is significantly better than that of GF(2) for poor channel conditions or high transmission redundancy. To improve the transmission reliability, we should let a relatively larger Galois field be used in SNC.

## 6. Performance Evaluation

In this section, we evaluate the performance of the proposed CLOR protocol by OPNET software [61] and compare it with two cross-layer protocols—CARP and FBR—and an enhanced flooding-based protocol (EFlood).

### 6.1. Simulation Scenarios and Settings

We consider a shallow sea located in 26.5 N and 123.5 E, with an average depth of 124 m, 21 nodes (including a sink) randomly anchored in a 7 × 3 grid with a total region size of 1 km × 2 km × 124 m, and the sink located in the left of the area, and the average distance of each neighbor is about 400 m. Bellhop is used to calculate the path loss between each node in a given location. In addition, the $\delta_{p}^{2}$ is set to 5 dB to represent a time-varying channel condition in this sea area. w and s are set to 6.5 m/s and to 0.5, respectively.

We assume that each node is equipped with a WHOI micromodem-2 [37,62]. Additionally, uncoded BPSK modulation is chosen to obtain a higher data rate. We select a carrier frequency of 25.6 kHz with a bandwidth of 4 kHz. The data rate is set to 4000 bps, so the bandwidth efficiency is 1 bps/Hz. To model the energy consumption, we set the transmit/receive/idle power to 2.8/0.8/0.285 W and assume that FBR uses three levels of transmission power: 2.8 W, 8 W, and 35 W.

In the simulation, the traffic is generated according to a Possion process with network traffic rates of {0.01, 0.02, 0.04, 0.08, 0.1, 0.12, 0.16, 0.2} packets/min. The payload of a data packet is set to {128, 256, 512} Bytes (B). The total size of a data packet is given by its payload plus the size of headers in different layers. As in [5], the physical header overhead changes with the data rate and is dominated by 10 ms for synchronization. The MAC header overhead of the handshake-based protocols and of Eflood are set to 4 B and to 3 B. The size of a HELLO packet is set to 6 B. We set the size of PONG/ACK in CARP to 7/6 B according to [5]. The size of CTF/ACK in CLOR are set to 8/7 B. Considering the length of control packets, we set the maximum size of PING/RTF to 20 bytes. In both CLOR and CARP, unless otherwise specified, the maximum burst size is set to 8. The maximum retry times of the handshake-based protocols are set to 3. The cone aperture θ in FBR is set to 2π/3. When SL = 158 dB re uPa @ 1m source, the average hop count, the average of the maximum hop count and the average node degree are 2.30, 4.48, and 4.91, respectively. The simulation time of each run lasts 7200 s, and the average value of 50 runs in different scenarios with the 95% confidence interval is reported.

The metrics used in the evaluation are defined as follows:

Packet delivery ratio (PDR): The ratio of packets successfully received by the sink node to the packets sent by source nodes.

End-to-end delay: The time of a packet reaching the sink plus the generating time of the corresponding packet.

Goodput: The total bit successfully delivered to the sink divided by the simulation duration.

Energy per bit: The energy consumption of the network for successfully delivering a bit of data to the sink.

### 6.2. Simulation Results

We first study the impacts of several parameters on CLOR network performance, and then evaluate CLOR against two cross-layer protocols, FBR and CARP, and an enhanced flooding-based protocol, EFlood, in terms of PDR, latency, energy efficiency, and Goodput.

#### 6.2.1. Impact of Various Parameters

##### Impact of Source Level

Source level influences the network connectivity and the spatial reuse in the network. A lower source level reduces the transmission range, which in turn decreases the size of the candidate set and then degrades the transmission reliability. In contrast, each node requires transmitting a packet with more hops to the sink node with a low source level. We set $\lambda_{T}$ = 2.0 packet/min, $N_{mBT}$ = 1, and $N_{MBT}$ = 8. Then, we simulate and analyze the impacts of source level on the network performance of CLOR protocol. 

**Figure 9:**
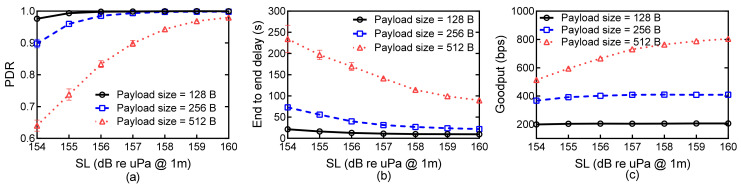
Network performances versus SL for different payload sizes: (**a**) PDR; (**b**) Average end-to-end delay; (**c**) Goodput.

The network topology changes slightly when SL increases from 154 dB re uPa @ 1m source to 160 dB re uPa @ 1m source. The average hop count decreases from 2.34 to 2.30 (the average of the maximum hop count decreases from 4.52 to 4.47), and the average node degree increases from 4.78 to 4.91. However, it heavily influences performance improvement of CLOR at large packet sizes. Figure 9 shows network performances versus SL for different payload sizes. As expected, the PDR of the scenarios with low SL is worse than that with high SL. Large payload size results in a large value of PER in the same channel condition, which degrades the transmission reliability. Thus, the PDR of the scenarios with a large payload size is significantly smaller than that with a small payload size for different values of SL. As shown in Figure 9b, the delay decreases significantly as SL increases, especially in the scenarios with large payload size. Higher SL results in a higher probability of network connectivity and a shorter average hop counts to the sink; this decreases the average end-to-end delay. As shown in Figure 9c, when the payload size is large, the Goodput of CLOR can reach an optimal value by setting a relatively high value of SL. The Goodput of payload size = 256 B is nearly two times that of that payload size = 128 B. This is same for the case that payload size = 512 B as compared with the networks with payload size = 256 B. This suggests that the network can achieve an optimal Goodput by increasing the value of SL to a relative high value.

##### Impact of Burst Size

As analyzed in Section 5, the burst transmission strategy effectively improves the transmission of CLOR. Meanwhile, the transmission reliability can be improved by using network coding in the burst transmission strategy. We set $\lambda_{T}$ = 2.0 packet/min and SL = 154 dB re uPa @ 1m source. Then, we simulate and analyze the impacts of the maximum and minimum burst size on the network performance of CLOR protocol.

**Figure 10:**
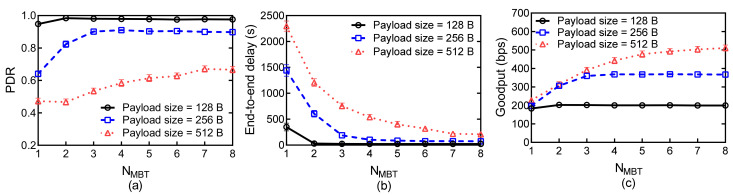
Network performances versus $N_{MBT}$ for different payload sizes with a fixed $N_{MBT}$: (**a**) PDR; (**b**) Average end-to-end delay; (**c**) Goodput.

Figure 10 shows the network performance for different maximum burst sizes $N_{MBT}$ and for different payload size with $N_{mBT}$ = 1. As shown in Figure 10a, the PDR increases as $N_{MBT}$ increases, and this improvement diminishes when $N_{MBT}$ reaches a certain value. As shown in Figure 10b, the delay performance can be improved with a large $N_{MBT}$. When $N_{MBT}$ = 1, the burst transmission degrades to normal transmission. CLOR seriously suffers from the collision problem, and the delay is significantly larger than that with a large $N_{MBT}$. As shown in Figure 10c, the Goodput increases and exceeds a certain value as $N_{MBT}$ increases. When payload size increases from 128 to 256 B, the Goodput only increases with a factor of 0.84. However, the Goodput can be improved with a factor of 0.39 when the payload size increase from 256 to 512 B. This suggests that the network cannot achieve an optimal Goodput by increasing the value of $N_{MBT}$ with a fixed $N_{mBT}$ in case of a small value of SL.

**Figure 11:**
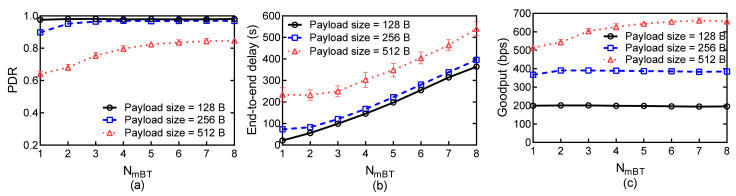
Network performances versus $N_{mBT}$ for different payload sizes with a fixed $N_{mBT}$: (**a**) PDR; (**b**) Average end-to-end delay; (**c**) Goodput.

Figure 11 shows the network performance for various minimum burst size $N_{mBT}$ and for different payload size with $N_{MBT}$ = 8. As shown in Figure 11a, the PDR increases as $N_{mBT}$ increases, and this improvement diminishes when $N_{mBT}$ reaches a certain value. By comparing Figure 10a with Figure 11a, we can find that $N_{MBT}$ influences the lower bound of PDR, whereas $N_{mBT}$ influences the upper bound of PDR. As shown in Figure 11b, the delay of the networks with different payload increases as $N_{mBT}$ increases. Since each nodes require queuing more packets to start a new handshake, the nodes with the number of packets in queues smaller than $N_{mBT}$ should wait extra time to execute transmission. This seriously influences the delay performance, especially with a large value of $N_{mBT}$. As shown in in Figure 10c and Figure 11c, the network can achieve high Goodput with relatively high values of $N_{mBT}$ and $N_{MBT}$ in case of large payload size.

#### 6.2.2. Comparison with Typical Protocols

**Figure 12:**
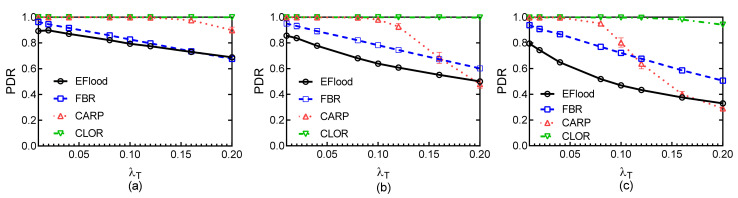
PDR: (**a**) Payload size = 128 B; (**b**) Payload size = 256 B; (**c**) Payload size = 512 B.

**Figure 13:**
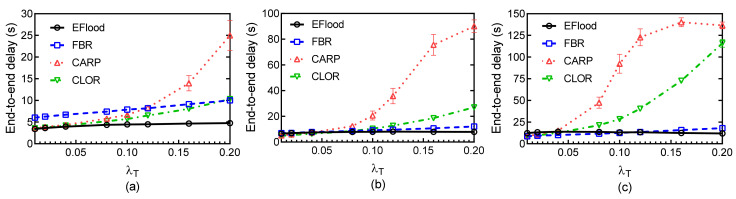
Average end-to-end delay: (**a**) Payload size = 128 B; (**b**) Payload size = 256 B; (**c**) Payload size = 512 B.

**Figure 14:**
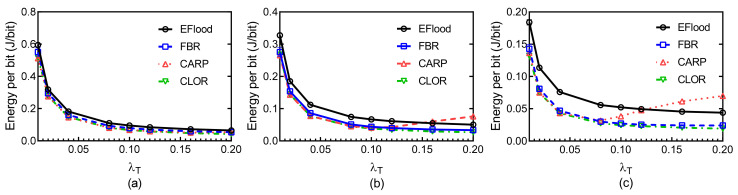
Energy efficiency: (**a**) Payload size = 128 B; (**b**) Payload size = 256 B; (**c**) Payload size = 512 B.

**Figure 15:**
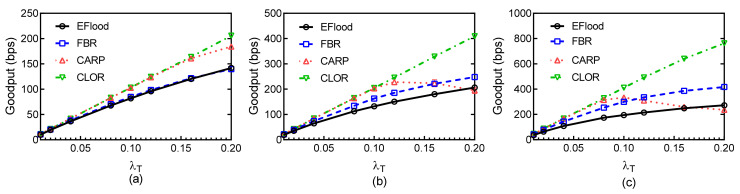
Goodput: (**a**) Payload size = 128 B; (**b**) Payload size = 256 B; (**c**) Payload size = 512 B.

Then, we simulate the four protocols for different traffic rates and for different payload sizes with $\lambda_{T}$ = 2.0 packet/min, SL = 158 dB re uPa @ 1m source, $N_{mBT}$= 1, and $N_{MBT}$ = 8. The comparison of CLOR against other typical protocols is shown in Figure 12, Figure 13, Figure 14 and Figure 15.

Figure 12 shows the PDR of the four routing protocols for different traffic rates $\lambda_{T}$ and for different payload sizes. The PDR of CLOR is significantly better than that of EFlood and FBR protocols, and it is better than CARP at high traffic rates when the payload size is large. At low traffic rates, Eflood shows the worst performance among the four protocols. All three handshake-based protocols outperform EFlood, and the difference of PDR of these three protocols are small. In EFlood, a node directly forwards a packet without handshaking with the surround nodes. However, this routing method cannot avoid the collision. In contrast, another three protocols use the handshake method to avoid the collision.

The PDR for the four protocols decreases as the traffic rate increases, especially when the payload is large, because the collision and the interference significantly increase as the traffic rate increases. In addition, the probability of the collision exponential increases as the payload size linearly increases, and this is especially obvious when the traffic rate is high. EFlood still suffers from the collision problem as the traffic rate increases. FBR can avoid collision at lower traffic rates. When the traffic rate increases, the probability of the successful ratio for the handshake that is triggered by a node decreases, and this will lead to the node dropping more packets than that at low traffic rates. In contrast, the burst transmission strategy used in CARP and CLOR can improve channel utilization and then decrease the probability of the collision in a network. Moreover, the transmission reliability can be further improved by the adaptive transmission strategy used in CLOR. 

At high traffic rates, the PDR of other three protocols decreases while CLOR still outperforms the other three protocols. The PDR of CARP decreases more sharply than the other three protocols for a large payload size, because the handshake strategy can effectively decrease the probability of the collision at small or middle traffic rates. At high traffic rates, the burst size significantly increases, and the transmission time is long for a large payload size. This leads to large transmission delay in one transmission. Then, the probability of the successful ratio of the handshake decreases, and more packets are dropped because of handshake failure. In CLOR, the back-off strategy used in the negotiation phase can effectively avoid the collision that is introduced by the handshake, and thus, CLOR is slightly influenced by high traffic rates for different payload sizes. Moreover, the congestion that is considered in the candidate set selection can further decrease the probability of the collision. In addition, the use of coding strategy in burst transmission can help to resist the influence of the bad channel condition since more multi-hop interference is introduced at high traffic rates.

Figure 13 shows the average end-to-end delay of the four routing protocols at different traffic rates $\lambda_{T}$ and for different payload sizes. EFlood outperforms the other three protocols at various traffic rates for different payload sizes, since each packet is forwarded directly without a handshake strategy in EFlood. At low traffic rates, the delay performance of CARP is better than FBR due to the effectiveness of the burst transmission strategy. CLOR has better transmission reliability than that of CARP due to the coding strategy in the burst transmission process. Thus, more packets in CLOR can be recovered than that in CARP. Less packets are required to be retransmitted in CLOR than that in CARP, since the packet may be sent by the sender with more times. Then, the average end-to-end delay of a packet in CLOR is smaller than that in CARP. 

When the payload size is small, the delay performance of CARP degrades heavily as the traffic rate increases, whereas the delay performance of CLOR is slightly increased at high traffic rates, because the transmission strategy is heavily influenced by the collision that is increased at higher traffic rates. In CLOR, the back-off strategy can handle the collision introduced by the negotiation. However, the time interval of a node in the BACKOFF state increases significantly with large payload size. Thus, the delay performance of CLOR degrades sharply with large payload size compared with that with small payload size for different traffic rates. Nevertheless, CLOR still outperforms CARP at high traffic rates.

Figure 14 shows the energy efficiency of the four routing protocols at different traffic rates $\lambda_{T}$ and for different payload sizes. As expected, three handshake-based protocols show better energy efficiency than that of EFlood. The energy efficiency of the three handshake-based protocols is close, because the inherent characteristics of redundancy transmission in EFlood introduce more energy for delivering a packet. At high traffic rates, EFlood heavily suffers from the collision problem, and more energy is wasted in flooding, which results in the ineffectiveness of energy performance. 

Figure 15 shows the Goodput of the four routing protocols at different traffic rates $\lambda_{T}$ and for different payload sizes. At low traffic rates, the Goodput of the four protocols are closed. As the traffic rate increases, CARP and CLOR perform approximately the same and outperform other two protocols. This is because the burst transmission strategy increases transmission effectiveness. We can find that the Goodput values of the three handshake-based protocols obviously outperform EFlood, because the collision heavily influences the transmission reliability of the protocols based on flooding. The probability of collision increases heavily as the payload size increases, which in turn significantly influences the transmission efficiency of EFlood. When the payload size is 512 B, the Goodput of CARP slightly decreases at high traffic rates, whereas the Goodput of the other three protocols still increases. This suggests that the probability of collision is large enough at high traffic rates and heavily influences the effectiveness of burst transmission in CARP. However, CLOR outperforms the other three protocols for different conditions as well.

## 7. Conclusions

In this paper, a Cross-Layer-Aided Opportunistic Routing Protocol is proposed to reduce the congestion in multi-hop sparse UWSNs. The forwarder selection algorithm based on a fuzzy logic is first presented to select an optimal forwarder with good forward link quality and small probability of congestion. On this basis, to alleviate the collision problem, a back-off strategy is introduced in CLOR. Moreover, an adaptive transmission strategy based on inter-session network coding is adopted to improve the transmission reliability of the data packets from various sources. Furthermore, this paper theoretically analyzes the main strategies in CLOR, including the effectiveness of negotiation, effectiveness of burst transmission, and reliability of network coding. Eventually, we employ the Bellhop ray tracer to model an underwater acoustic channel of a specific sea region, and we compare the performance of CLOR with the existing Eflood, FBR, and CARP in the networks under different traffic rates. Simulation results indicate that the proposed CLOR outperforms other three protocols in terms of packet delivery ratio, energy efficiency, and Goodput at different traffic rates, especially at high traffic rates, while CLOR also performs outstanding advantages in an aspect of end-to-end delay at low traffic rates. The future work will be focused on improving the processing procedure of CLOR and further minimizing the delay of CLOR.

## Data Availability

Not applicable.
